# Potent and selective effect of the mir-10b inhibitor MN-anti-mir10b in human cancer cells of diverse primary disease origin

**DOI:** 10.1371/journal.pone.0201046

**Published:** 2018-07-20

**Authors:** Byunghee Yoo, Patricia Greninger, Giovanna T. Stein, Regina K. Egan, Joseph McClanaghan, Anna Moore, Cyril H. Benes, Zdravka Medarova

**Affiliations:** 1 Molecular Imaging Laboratory, MGH/MIT/HMS Athinoula A. Martinos Center for Biomedical Imaging, Massachusetts General Hospital and Harvard Medical School, Boston, MA, United States of America; 2 Center for Molecular Therapeutics, Cancer Center, Massachusetts General Hospital and Harvard Medical School, Boston, MA, United States of America; University of South Alabama Mitchell Cancer Institute, UNITED STATES

## Abstract

Since microRNAs (miRNAs, miRs) have been implicated in oncogenesis, many of them have been identified as therapeutic targets. Previously we have demonstrated that miRNA-10b acts as a master regulator of the viability of metastatic tumor cells and represents a target for therapeutic intervention. We designed and synthesized an inhibitor of miR-10b, termed MN-anti-miR10b. We showed that treatment with MN-anti-miR10b led to durable regression/elimination of established metastases in murine models of metastatic breast cancer. Since miRNA-10b has been associated with various metastatic and non-metastatic cancers, in the present study, we investigated the effect of MN-anti-miR10b in a panel of over 600 cell lines derived from a variety of human malignancies. We observed an effect on the viability of multiple cell lines within each cancer type and a mostly dichotomous response with cell lines either strongly responsive to MN-anti-miR10b or not at all even at maximum dose tested, suggesting a very high specificity of the effect. Genomic modeling of the drug response showed enrichment of genes associated with the proto-oncogene, c-Jun.

## Introduction

Systemic treatment options for cancer often include chemotherapy, as a mainstream treatment approach. However, considering the disadvantages of standard chemotherapy, e.g. non-specific delivery, toxicity to healthy tissues, distressing side effects (including fatigue, nausea, vomiting, and hair loss), and the potential for chemoresistance, we envision a future in which chemotherapy is complemented or replaced by alternative approaches to shorten the period of chemotherapy or lower the dose of anti-cancer drugs and minimize adverse symptoms while increasing the survival rate.

The alternative that we propose relies on targeting oncogenic microRNAs (oncomiRs) that promote the migration, proliferation, and survival of tumor cells. In our earlier work, we identified miRNA-10b as a master regulator of the viability of metastatic tumor cells. We determined that miR-10b not only promotes the ability of tumor cells to migrate and invade surrounding tissue (become metastatic), as has been described previously [1, 2] but, most importantly, serves as a powerful master regulator of the viability of these cells [3, 4]. Detailed mechanistic studies confirmed the existence of a miR-10b-triggered pathway that regulates the viability and proliferation of tumor cells in the metastatic microenvironment. This knowledge allowed us to develop a therapeutic strategy based on miR-10b inhibition. The specific inhibition of miR-10b was achieved using inhibitory oligonucleotides (LNA-based antagomirs) delivered to metastatic sites by dextran-coated iron oxide nanoparticles (termed MN-anti-miR10b) [5].

We demonstrated that MN-anti-miR10b could completely prevent the formation of de novo metastases [3] and, when combined with low-dose chemotherapy, caused complete and persistent regression of local lymph node metastases in the MDA-MB-231 breast cancer model with no evidence of systemic toxicity [4].

In a model of Stage IV metastatic breast cancer, we found that MN-anti-miR10b selectively accumulated in distant (lung, bone, and brain) metastases from breast cancer after intravenous injection. A weekly treatment protocol with MN-anti-miR-10b and low-dose doxorubicin demonstrated complete regression of pre-existing lung metastases in 65% of the animals and inhibition of multiple organ metastases in 94% of the animals. This translated into a significant reduction in cancer mortality in animals treated with MN-anti-miR10b and low-dose doxorubicin relative to control groups, including a group treated with monotherapy of standard dose doxorubicin, used to model standard-of-care.

miRNA-10b has been implicated in the development of a wide range of human cancers including colorectal, gastric, bladder, pancreatic, ovarian, hepatocellular and brain cancer [6–8]. Recent studies have linked microRNA-10b to migration, invasion, cell viability, and proliferation in non-small cell lung cancer (NSCLC) [9] and cervical cancer [10], and have suggested new roles for miR-10b in oncogene-induced tumorigenesis and metastasis through inhibition of tumor-suppressive mechanisms in mammary carcinoma [11]. Based on the prior knowledge about the broad influence of miR-10b on a number of human malignancies, in this study we embarked on a systematic investigation of its global effect on cancer. Our approach is based on our discovery that in addition to influencing invasion and migration of tumor cells, miRNA-10b is responsible for the survival of metastatic cells [4]. For that reason, the present study focused on investigation of tumor cell viability following miR-10b inhibition using MN-anti-miR10b in a panel of representative cell lines including metastatic and non-metastatic cancers. We found a distinct pattern of strong responses in all cancer types.

Our study revealed the association of these responses with gene expression patterns that suggested an effect on transcription, metastatic dissemination, and immune response. Finally, our data implied that the link between miR-10b inhibition and cell viability is not limited to metastatic cells, but also includes cells derived from primary tumors, which is a novel finding. We believe that this finding could have a significant impact on treating both non-metastatic and metastatic cancers.

## Materials and methods

### MN-anti-miR10b synthesis and characterization

MN-anti-miR10b was synthesized as described in [12]. The LNA antagomirs used in this study were designed and synthesized by Exiqon Inc. (Woburn, MA). The 5’-Thiol-Modifier C6 disulfide (5’-ThioMC6) was inserted into the anti-miR10b oligo for conjugation to magnetic nanoparticles [3, 13].

Aminated magnetic nanoparticles were synthesized following a protocol published previously (1, 2). Nanoparticles with a size of 20.3 ± 0.6 nm were used for conjugation to the oligonucleotides. The magnetic nanoparticles were conjugated to the heterobifunctional linker N-succinimidyl 3-[2-pyridyldithio]-propionate (SPDP; Thermo Scientific Co., Rockford, IL) and activated oligos sequentially. Briefly, SPDP was dissolved in anhydrous DMSO and incubated with magnetic nanoparticles. The 5’-ThioMC6 of the oligo was activated to release the thiol via 3% TCEP treatment in nuclease-free PBS. The oligos were purified using an ammonium acetate/ethanol precipitation method. After TCEP activation and purification, the oligos were dissolved in water and incubated with the SPDP-modified magnetic nanoparticles overnight. The number of oligos per magnetic nanoparticle was determined as 8.0 ± 0.7 using the electrophoresis analysis method described previously [3, 13].

### Cell line collection

We have assembled a collection of 624 human tumor cell lines representing the spectrum of common and rare types of adult and childhood metastatic and non-metastatic cancers of epithelial, mesenchymal and haematopoietic origin. We have used this large panel of cell lines in order to better capture the high degree of genomic diversity in cancer and to identify rare mutant subsets with altered sensitivity. All cell lines were obtained from commercial repositories. Cell lines were expanded for less than 3 passages and frozen stocks were created. To further test the quality of the collection post expansion we performed the following: To identify cross-contaminated or synonymous lines, a panel of single nucleotide polymorphisms (SNPs) was profiled for each cell line (Sequenom, San Diego, CA) and a pair-wise comparison score calculated. In addition, we performed short tandem repeat (STR) analysis (AmpFlSTR Identifiler, Applied Biosystems, Carlsbad, CA) and matched this to an existing STR profile generated by the providing repository. Cells from authenticated stocks were not continuously kept in culture for more than 3 months.

### Cell viability assays

Cells were seeded in 384-well microplates at ~15%-50% confluence in medium with 10% FBS and penicillin/streptavidin + high glucose (18–25 mM). The optimal cell number for each cell line was determined to optimize growth during treatment. For adherent cell lines, after overnight incubation, cells were treated with increasing concentrations of MN-anti-miR10b (2-fold dilution series ranging from 0.16 to 10.64 μM as oligo) using liquid handling robotics, and then returned to the incubator for assay at a 72-hr time point. For suspension cell lines, cells were treated with MN-anti-miR10b immediately following plating and returned to the incubator for 72 hrs. Cells were then stained with 55 μg/ml Resazurin (Sigma) prepared in Glutathione-free media for 4 hours. Quantitation of fluorescent signal intensity was performed using a fluorescence plate reader at excitation and emission wavelengths of 535/595 nM for Resazurin. All screened plates were subjected to stringent quality control measures that included having coefficient of variance of control well values below 20% and signal over noise (control, untreated wells over blank wells) over 5-fold. Additionally, directionality of drug response was tested by comparing the average of viability values obtained with the two minimum doses and the two maximum doses and rejecting drug response if the ratio was below 1.2 (wrong direction of response). Effects on cell viability were measured and a curve-fitting algorithm was applied to this raw dataset to derive a multi-parameter description of drug response, including the half maximal inhibitory concentration (IC50). Curve fitting was performed using the drexplorer R package [14].

### Genomic modeling

The linear regression Elastic Net (EN, a multivariate linear regression approach) was used to obtain a multivariate model of the drug response across all cell line tested. We used Tissue of origin (Tis), Copy Number (CN), RNA expression data and mutation (MUT) data as input together with IC50s. RNA expression was U219 Affymetrix chip based. To minimize the number of genomic features in the input, the CN data was restricted to 1700 genes for which copy number alterations were translated into RNA expression. Mutation data were from full Exome Seq after algorithmic filtering of germline events (no matched germline sequence available for the cell lines). The EN modeling was run 100 times with 10% of the cell lines randomly left out in each run (leave 10% out cross-validation). To identify biological mechanisms related to the genes identified as response predictor by EN we used the STRING (v 10.5; 0.4 –evidence confidence setting) database [15] on the full list of genes associated with negative weight in the EN output (high expression overall in sensitive cell lines).

## Results

### MN-anti-miR10b elicits strong viability responses in a distinct subset of cell lines

**Fig 1:**
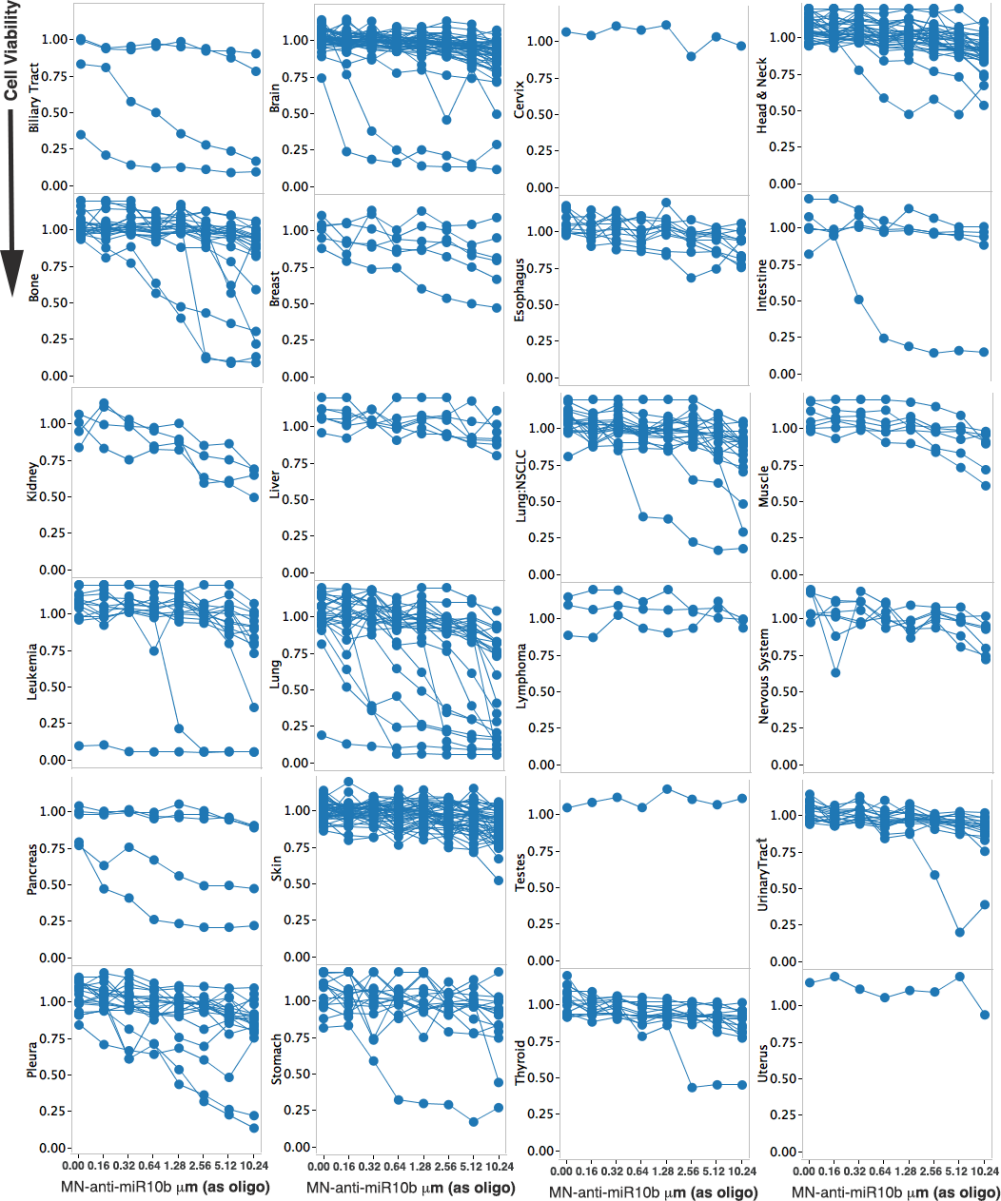
Cell viability. Representative line graphs of MN-anti-miR10b concentration vs cell viability for multiple cell lines within representative cancer tissues of origin.

**Fig 2:**
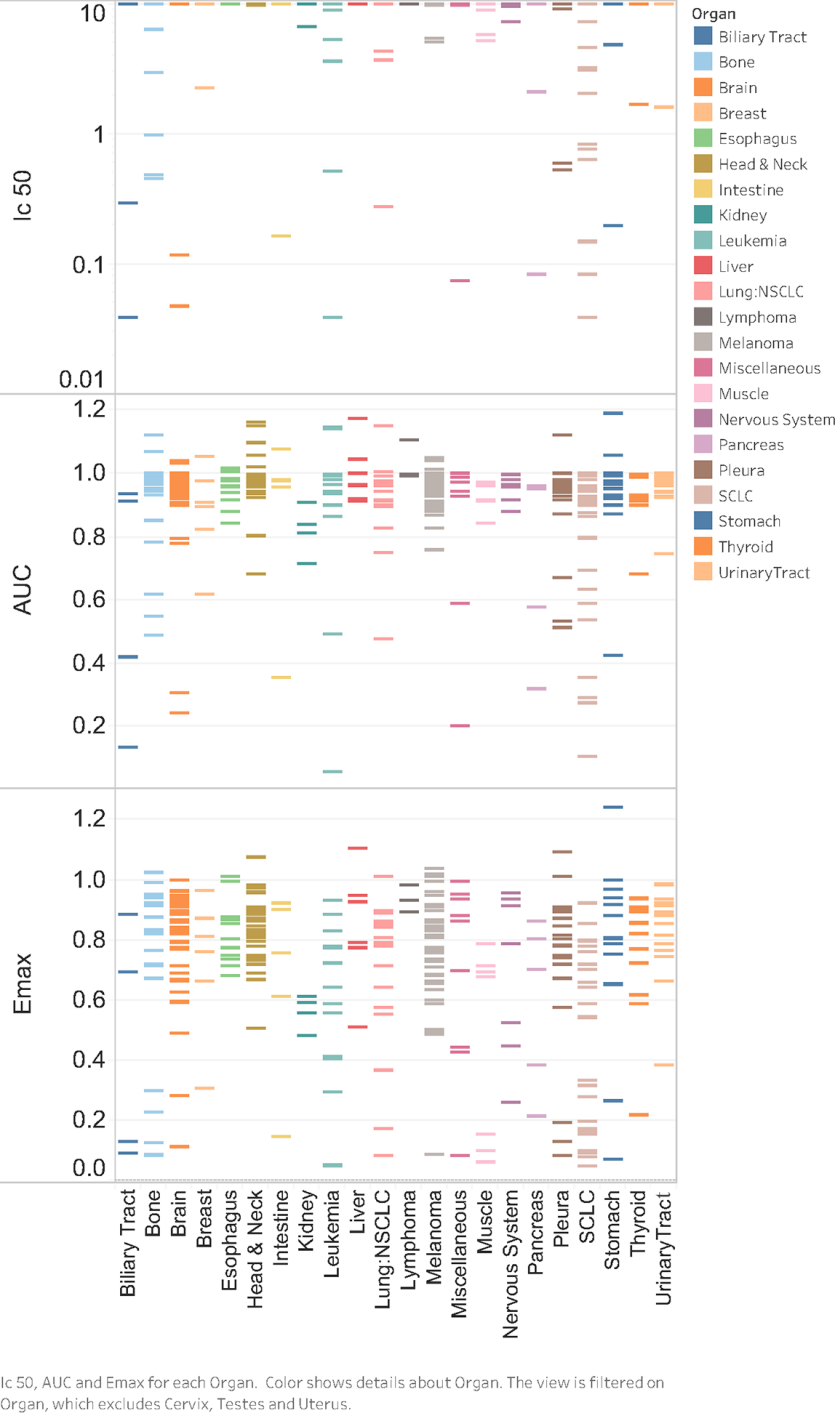
Profile of response across cell lines from different tissues of origin. The response to MN-anti-miR10b is shown as IC50 (μM), Area Under the dose response Curve (AUC) or Emax (maximum effect observed: minimum cell viability observed across the two maximum doses tested).

The sensitivity to MN-anti-miR10b was tested in 624 human cell lines representing metastatic and non-metastatic cancers of the following origin: Biliary Tract, Bone, Brain, Breast, Esophagus, Head & Neck, Intestine, Kidney, Leukemia, Liver, Lung, Lymphoma, Muscle, Nervous System, Ovary, Pancreas, Pleura, Skin, Stomach, Thyroid, Urinary Tract, and Uterus. Representative data are included in Figs 1 and 2. Strong responses could be seen in a distinct subset of cell lines from all of the cancers that were tested. Interestingly, we observed essentially two types of response with cell lines either strongly responsive to MN-anti-miR10b or not at all even at maximum dose tested. This is consistent with a very high specificity of the effect and shows that a distinct group of cell lines are highly sensitive to suppression of miR10b in several cancer types. Note that in some cases the number of models were small, such as in cervical and testicular cancer cell lines, and thus lack of response was not conclusive (Figs 1 and 2 and S1 Dataset).

### The responses to MN-anti-miR10b demonstrate meaningful functional relationships to genes identified by genomic modeling

The Genomic Modeling results are presented in S1 Dataset. In the Elastic Net modeling output the Freq corresponds to the frequency at which a given feature (gene) is present across the 100 modeling iterations using 10% left out cross-validation. A Freq of 1 indicates that all (100) models run contained that feature. The weight is the weight associated with each feature in the linear regression model (effect is a normalized weight taking into account the number of features of each type). Genes associated with negative weight values correspond to presence of mutation, amplification or higher relative mRNA expression in sensitive cell lines overall (not all sensitive cell lines need to present with all features as this is a multivariate approach across response of all the cell lines). For CN features a negative value means that there are overall more copies of that gene in the sensitive lines. Positive effects correspond to resistance. For the cells that have a high expression of these genes, the IC50 values tend to be higher.

Genes associated with resistance that were present at a frequency of > 0.7 included:

HEXB (responsible for the degradation of GM2 gangliosides, and a variety of other molecules containing terminal N-acetyl hexosamines, in the brain and other tissues);

ITPRIPL2 (encodes a membrane-associated protein that binds the inositol 1,4,5-trisphosphate receptor, ITPR. The encoded protein enhances the sensitivity of ITPR to intracellular calcium signaling. ITPRs have been found to regulate autophagy in normal cells and are associated with metastasis);

SEPW1 (plays a role as a glutathione (GSH)-dependent antioxidant and may be involved in a redox-related process and cell cycle progression); and

RPN2 (involved in translocation and the maintenance of the structural uniqueness of the rough ER. It is also an essential subunit of N-oligosaccharyl transferase complex that conjugates high mannose oligosaccharides to asparagine residues in the N-X-S/T consensus motif of nascent polypeptide chains. RPN2 has been demonstrated to be a prognostic marker of human cancer, is highly expressed in cancer stem cells and associated with metastasis).

Genes associated with sensitivity that were present at a frequency of > 0.7 included:

VPREB3 (a gene involved in B cell maturation);

ZBTB44 (a zinc finger protein involved in nucleic acid binding);

BCL7A (a tumor-suppressor, possibly anti-apoptotic gene);

PTPN18 (a protein tyrosine phosphatase, commonly involved in cell growth, differentiation, mitotic cycle, and oncogenic transformation);

VPREB1 (a gene that belongs to the immunoglobulin superfamily and involved in early stages of B cell development);

LTB (member of the TNF family, and inducer of the inflammatory response);

SARNP (possibly involved in cell cycle progression);

CD79B (encodes the Ig-beta protein of the B cell antigen component); SPIB (a transcriptional activator);

APBB1IP (appears to function in the signal transduction from Ras activation to actin cytoskeletal remodeling; suppresses insulin-induced promoter activities through AP1 and SRE and mediates Rap1-induced adhesion);

ANKLE1 (endonuclease that probably plays a role in the DNA damage response and DNA repair); and

LIMD2 (acts as an activator of the protein-kinase ILK, thereby regulating cell motility) [S1 Dataset].

Annotation of the genomic modeling output using STRING to identify functional networks and enrichment of biological processes revealed that the genes associated with sensitivity present with more connection in STRING than expected by chance (Expected number of connection 47, observed number 96; p = 3.10^−10^) This is supportive of a meaningful functional relation between the genes identified by EN modeling. The list of genes associated with sensitivity (negative weight values in the EN) was enriched for genes involved in chromatin organization (GO:0006325; FDR<8.10^−5^) and overall for gene products located in the nucleus (GO:0005634; FDR = 0.05). The genes associated with resistance were also more interconnected (based on STRING) than expected by chance (expected number of connections 18, observed number of connections 72; FDR = 0). The top biological processes enriched were extracellular matrix organization (FDR = 0.0002), and endomembrane system localization (GO:0012505, FDR<2.10^−9^). Members of the lysosome pathway (KEGG 04142) were also enriched in the genes more expressed in resistant cell lines (FDR<6.10^−5^). A full list of enrichment terms and values can be found in S2 Dataset.

**Fig 3:**
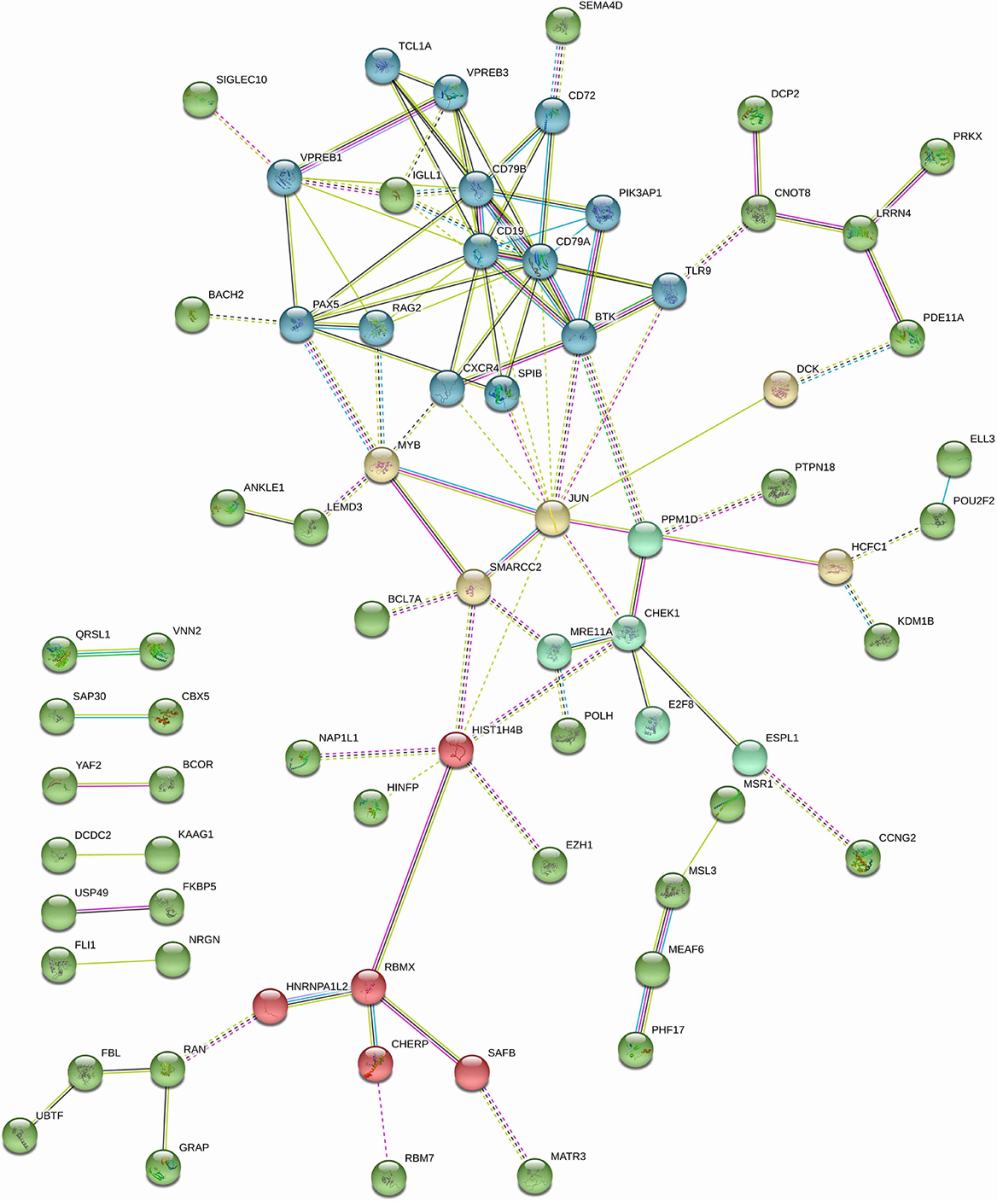
Network of proteins encoded by the genes found to be predictive of sensitivity to MN-anti-miR10b by elastic net regression together with JUN. The functional network was built in STRING and kmeans clustering was performed to identify subnetworks (set to 5 clusters in STRING using all evidence of interactions and interaction score of 0.4 or more). Nodes from the EN output that were not found to be connected are excluded for visualization purposes.

Based on a recent publication linking miR10b to the control of c-Jun [16] we thought to investigate functional connections between c-Jun and genes identified as predictor of response. We included c-Jun together with the list of genes identified by EN and performed a new STRING analysis. Interestingly, several of the genes associated with sensitivity have a known connection to c-Jun. Among those MYB, SMARCC2, SPIB, DCK, PPM1D, CHEK1 represent candidate modulators of the effect of MN-anti-miR10b. A STRING derived network of genes found to be predictive to the response to MN-anti-miR10b and their known relation to c-Jun is presented in Fig 3.

## Discussion

In 2007, Ma and colleagues for the first time demonstrated aberrant expression of miR-10b in cancer [1, 2]. Since then the role of miR-10b as a metastasis promoting factor has been uncovered. To date, more than 100 studies have been completed on miR-10b and metastasis across 18 cancer types. miR-10b has been correlated with progression to metastasis in 13 out of 18 types of cancer studied, including bladder, breast, cervical, colorectal, gastric, hepatocellular, lung, melanoma, nasopharyngeal, osteosarcoma, neuroblastoma, thyroid, and pancreatic cancer [1, 17–43]. This large body of evidence suggests that it is feasible to achieve powerful therapeutic effects across many human cancers by inhibiting miR-10b. Still, despite the strong association between miR-10b expression and cancer progression, there is a limited understanding of the precise mechanisms by which miR-10b exerts its effect in different cancers. Besides a clear influence on tumor cell migration and invasion in most cancers, miR-10b has shown association with tumor cell viability and proliferation [4, 5], immune evasion [29], angiogenesis [30], and stem cell differentiation [31]. However, these results have been limited by cancer type and have not involved systematic analysis that would permit the identification of global effects that are independent of cancer type.

Our own early results strongly indicated that miR-10b expression is essential for tumor cell survival, and if this miRNA is inhibited, the cells lose their viability. Still, the biology behind this discovery needed to be explained since previously miR-10b was only implicated in invasion and migration of cancer cells but not in viability or proliferation [1, 2].

A recent publication independently elucidated the precise mechanism explaining our observations [16]. Namely, it was shown that at the receiving end of the miR-10b pathway is the proto-oncogene c-Jun, a transcription factor that plays a critical role in stimulation of cell proliferation and tumor progression. Interestingly, c-Jun is translationally activated by loss of cell contacts or restructuring of the cytoskeleton–a process specific to the metastatic tumor cell and characterized by loss of E-cadherin expression. This new knowledge explains our observation that the pro-apoptotic effect resulting from miR-10b inhibition is linked to the metastatic phenotype.

Based on this, the following model is proposed: in the course of uncontrolled primary tumor growth, isolated pockets within the primary tumor microenvironment emerge that are characterized by poor cell-cell and cell-substrate contacts. Within these pockets, cytoskeletal restructuring and loss of E-cadherin occur, in response to the newly acquired need for anchorage-independent growth. These changes trigger increases in miR-10b expression as an adaptive process that ensures the survival of these cells. Because of the direct link between miR-10b overexpression and the pro-metastatic HOXD10, these miR-10b overexpressing cells are not only able to survive in the absence of proper cell-cell and cell-stroma contacts but are also capable of initiating the key steps necessary for traveling to distant sites and colonizing them. This model explains why the inhibition of miR-10b in metastatic cells using MN-anti-miR10b leads to loss of viability.

At the same time, we found that the loss of cell viability after treatment with MN-anti-miR10b is not limited to metastatic cells. It is possible that this miRNA is also highly expressed in rapidly proliferating cells from aggressive cancers, and its inhibition causes a similar chain of events leading to loss of viability. The precise mechanism behind this observation is currently under investigation.

Within this context, in the present study we embarked on a systematic investigation that would help us gain insight into the general mechanisms by which miR-10b exerts its effect in human cancer. The results presented here not only confirmed the role played by miR-10b but also suggested a link between miR-10b and genes involved in chromatin organization, extracellular matrix organization, endomembrane system localization, and lysosome function. Importantly, these associations are found across multiple cancer types and reflect a fundamental role of miR-10b in cancer biology. Notable is the enrichment of genes associated with the proto-oncogene, c-Jun. c-Jun is a key regulator of cell cycle progression and inhibitor of apoptosis. It plays a crucial role in tumor initiation and progression and is linked to both primary and metastatic cancers. By identifying new pathways that define the response to MN-anti-miR10b and providing further support for the link between miR-10b and c-Jun, the current studies expand our understanding of the array of phenotypic effects associated with this important therapeutic target.

## Supporting information

S1 Dataset Linear regression Elastic Net results. The linear regression Elastic Net was used to obtain a multivariate model of the drug response across all cell line tested. We used Tissue of origin (Tis), Copy Number (CN), RNA expression data and mutation (MUT) data as input together with IC50s. Genes are stratified based on sensitivity to MN-anti-miR10b.
(XLSX)

S2 Dataset Linear regression Elastic Net results. The linear regression Elastic Net results were stratified according to their enrichment score and grouped by annotation to arrive at common biological processes explanatory for sensitivity to MN-anti-miR10b.
(XLSX)
